# Homœopathy, as Regarded by One of Its Leaders

**Published:** 1887-03

**Authors:** 


### *HOMŒOPATHY, AS REGARDED BY ONE OF ITS LEADERS.*

Jousset, of Paris, is unquestionably one of the lights of homœopathy on the Continent of Europe. His recently published *Lecons de Clinique Médicale* is in some respects a model of its kind. According to this authority, the homœopath of to-day no longer affirms the mysterious potency of the globule, or the all-sufficiency of the doctrine of similars, but claims to be, in the true sense of the word, eclectic.

"Hahnemann and his pupils," he says, "pretended that homœopathy was the *whole* of therapeutics. This is a complete misconception of the case—homœopathy is but a *part* of therapeutics; this is a ruth which has cost us many execrations from men in our own ranks, but is now held to be indisputable.

"The fact is, homœopathy cannot take the place of *palliative* medication; nor of *surgical* medication; nor of *antidotal* medication in cases of poisoning; nor of *parasiticide* medication, wherever clearly demanded; nor of medication by *mineral waters*, which often cures where other modes of treatment fail; nor of *hydro-therapeutic* medication; nor of medication by electricity; nor even altogether of *empirical* medication. Homœopathy is not everything, and liberal medicine must include all collateral modes of treatment."

Jousset repudiates the allegation that homœopathy is a *sect*, and affirms that it is simply a branch of medicine which has to do with the therapeutics of certain internal disorders, and not even all of these are amenable to treatment by the law of similars (for example, helminthic diseases). The same writer, who seems to have some following in France, and may be said to represent the advanced thought of his school, gives some pretty hard blows at the advocates of infinitesimal doses, who, he intimates, have brought discredit upon homœopathy, and affirms that "the school of high dilutionists is losing ground every day, and in France, as in Germany and America, the general tendency is to employ the low dilutions."—*Boston Medical and Surgical Journal.*

